# Supplementary material for: High-Risk Lineages of Hybrid Plasmids Carrying Virulence and Carbapenemase Genes
Source: Antibiotics (Basel). 2024 Dec 17;13(12):1224. doi: 10.3390/antibiotics13121224 (PMC11726917; doi:10.3390/antibiotics13121224)

Supplementary Figure S1. Replicon diversity reported in each mge-cluster (n=18). The area of the plot is proportionally split into distinct tiles based on the number of plasmids. For each tile, the replicon combination is indicated in the center.

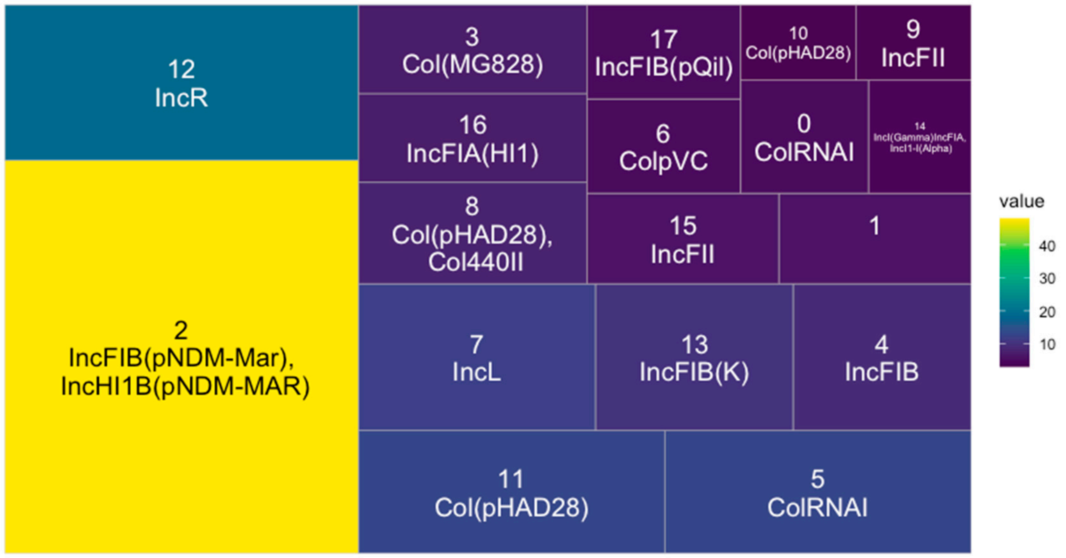

Supplementary Figure S2. Clustering of plasmids based on the mge-cluster analyses. (A) Each point corresponds to a plasmid sequence and is coloured (from red to blue) based on its membership probability. Unassigned sequences (plasmids with a membership probability of 0 of belonging to any defined cluster) are coloured in gray. The ellipses delimit the cluster coordinates and were estimated using the Khachiyan algorithm implemented in the ggforce R package. (B) Points (plasmids) are colored by presence/absence of ARGs. C – Points are colored by presence/absence of VFs.

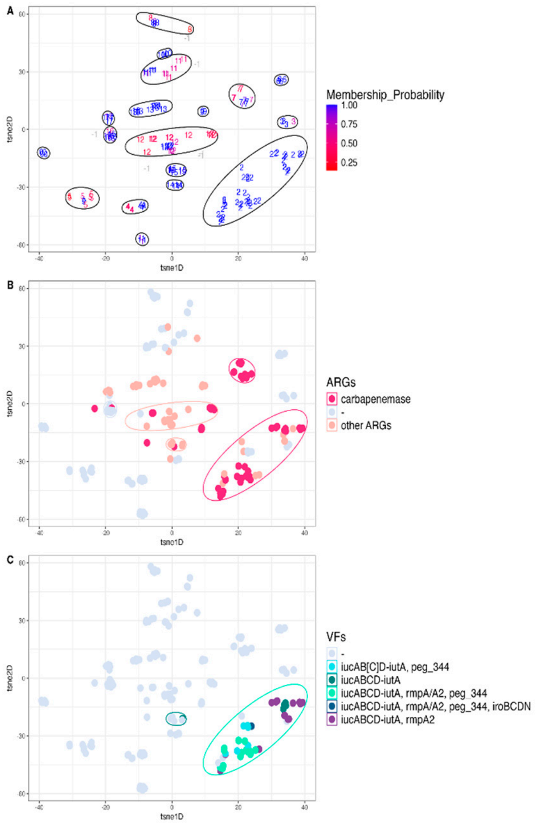

Supplementary Figure S3. Length and mobility of analyzed plasmids.

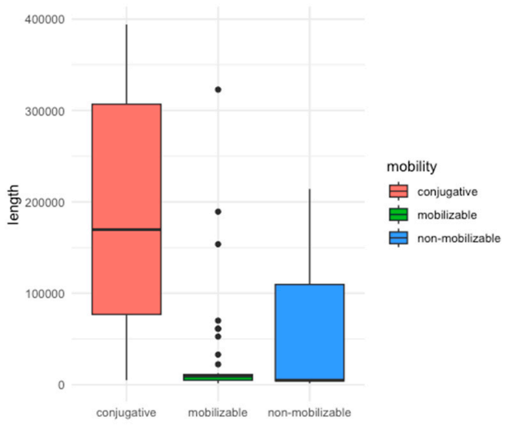

Supplementary Figure S4. Hierarchical clustering dendrogram of analyzed plasmids. The plasmids from cluster 2 were compared with highly similar plasmids downloaded from the NCBI database. In the legend, "this study" indicates the source of the sequence: either sequenced in this study (black) or downloaded from the NCBI database (white).The colors of the "cluster" legend show the assignment of each plasmid by cluster, whereas the "country" legend shows the geographical location of the isolation of the plasmid.

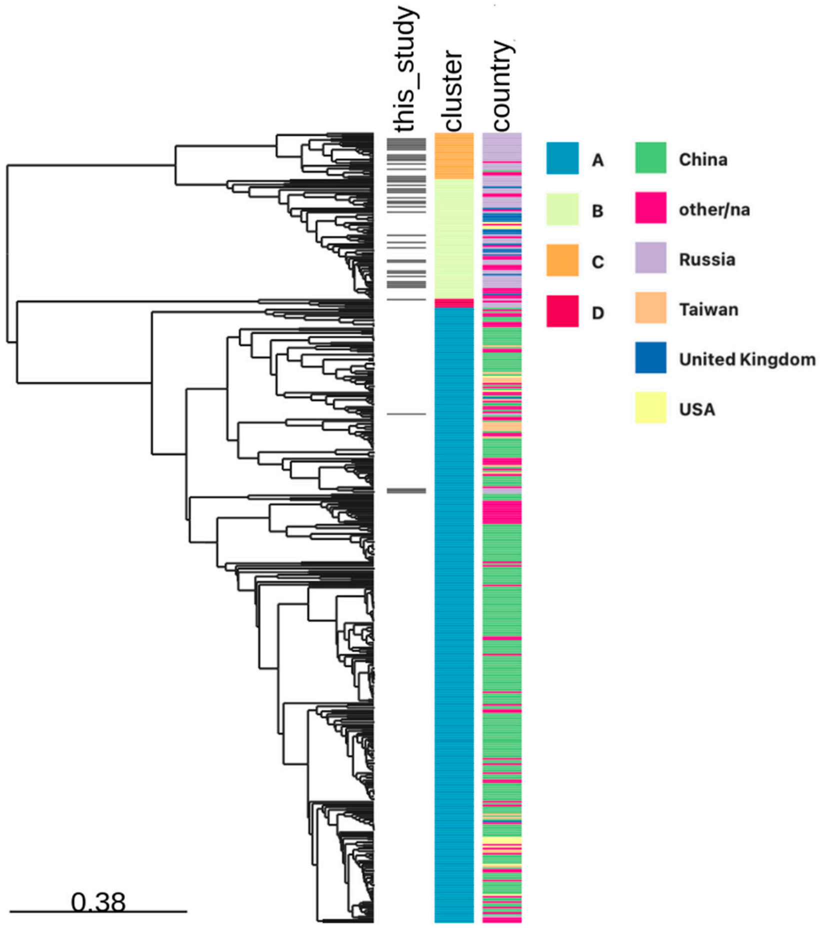

Supplementary Figure S5. Sequence similarity network of IncHI1B(pNDM-MAR)/ IncFIB(pNDM-Mar)-like plasmids from cluster 2 and the "Global dataset". Plasmids (nodes) are

connected with an edge where the unitig content using Jaccard coefficient is more than 0.8. A. Each node is colored by detected communities. B. Each node is colored by sequence type (ST) of the strain from which the plasmid was extracted.

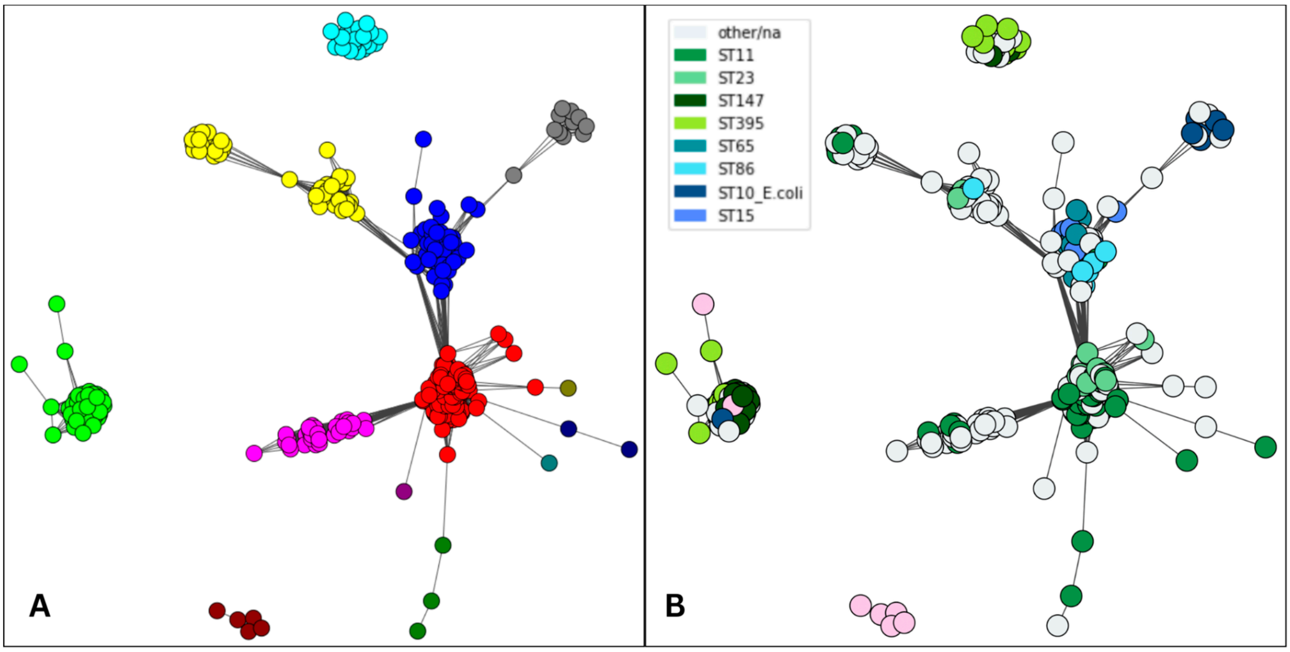

Supplement: Supplementary file 1 [file antibiotics-13-01224-s001.zip › supplementary_figures_6_12.pdf]
